# Scalable Fabrication and Testing Processes for Three-Layer Multi-Color Segmented Electrowetting Display

**DOI:** 10.3390/mi10050341

**Published:** 2019-05-23

**Authors:** Guisong Yang, Biao Tang, Dong Yuan, Alex Henzen, Guofu Zhou

**Affiliations:** 1 Guangdong Provincial Key Laboratory of Optical Information Materials and Technology & Institute of Electronic Paper Displays, South China Academy of Advanced Optoelectronics, South China Normal University, Guangzhou 510006, China; yangguisong@m.scnu.edu.cn (G.Y.); tangbiao@scnu.edu.cn (B.T.); alex.henzen@guohua-oet.com (A.H.); 2 National Center for International Research on Green Optoelectronics, South China Normal University, Guangzhou 510006, China; 3 Shenzhen Guohua Optoelectronics Tech. Co. Ltd., Shenzhen 518110, China; 4 Academy of Shenzhen Guohua Optoelectronics., Shenzhen 518110, China

**Keywords:** scalable fabrication and testing processes, multi-color, 3-layer segmented electrowetting display

## Abstract

Colorful electrowetting displays (EWD) present many challenges, such as scalability and electro-optical performance improvement (e.g., brightness, color gamut, and contrast ratio). The first full investigation of scalable fabrication and testing processes for multi-color segmented EWD with potentially unprecedented electro-optical performance is proposed. A three-layer architecture is employed to achieve colorful EWD, where the key components are three primary color layers (cyan, magenta, and yellow), switched independently. Unlike previous reports referred to herein, which used the same fabrication and testing processes for each layer, this architecture facilitates a uniform performance, improves yield, and simplifies the process for colorful EWD. With an aperture ratio greater than 80%, National Television Standards Committee (NTSC) color gamut area greater than 63%, switching speed lower than 12 ms, and DC driving voltage below 22V, the testing results of colorful EWD are proven successfully by using our proposed processes. The processes investigated in this paper have greatly improved efficiency, suitable for a high-volume of full-color EWD.

## 1. Introduction

Reflective displays utilize ambient light to illuminate the screen, thereby providing superior energy efficiency, sunlight readability, and reading comfort. These properties make them suitable for applications such as e-book readers, signage, electronic shelf labels, and portable paper-like display devices. Reflective displays such as electrowetting displays (EWD) [1,2,3], electrophoretic displays (EPD) [4], and cholesteric liquid crystal displays (LCD) [5] have been reported. One example of commercial success is the Amazon Kindle EPD e-reader, but EPD has a slow response time, which makes it impossible to play video smoothly. 

**Figure 1:**
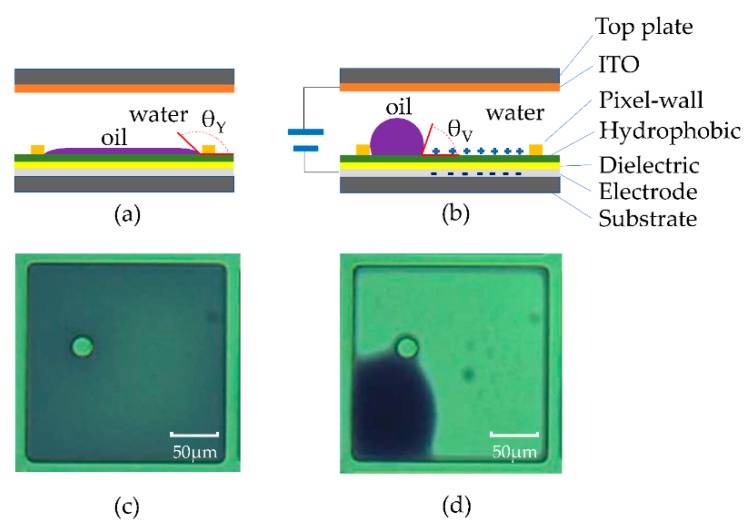
Schematic of electrowetting display and images of a tested sample. (**a**) Without voltage applied, a homogeneous oil film is present, showing the colored off-state. (**b**) With voltage applied, the oil film contracts, showing the white on-state. (**c**,**d**) The corresponding top view micrographs demonstrate a typical oil relaxation and contraction of a pixel. The size of the square pixel is 200 μm × 200 μm.

Robert A Hayes et al. first proposed an EWD to show the ability of video-speed response time, published in 2003 [1]. J. Heikenfeld used the Young–Laplace transposition of brilliant pigment dispersions to realize electro-fluidic displays showing its low power consumption in 2009 [2]. The white state reflectance of an EWD can reach >50% [6], it has also shown the potential for flexibility [7], and multi-gray scale [8]. Generally, the electro–optic behavior of EWDs is achieved by controlling the motion of two immiscible fluids, namely polar water and non-polar oil, in confined pixels, as recently reviewed and reported by Mugele [9]. A typical multi-layer pixel structure and the operating principles of EWDs are shown in Figure 1. The oil film completely covers the pixel substrate (off-state) at its Young’s contact angle ($\theta_{Y}$) and the pixel presents the color of the oil without applied voltage (Figure 1a,c). The oil is pushed aside by water and the observed contact angle reduces to $\theta_{V}$, showing the color of the substrate (on state) under the electrostatic field applied between the indium tin oxide (ITO) electrode on the top plate and substrate electrode (Figure 1b,d), according to [10]:

(1) $$\cos\theta_{V}=\cos\theta_{Y}+\frac{CV^{2}}{2\gamma_{AO}},$$

where C is the capacitance per unit area of the hydrophobic and dielectric (F/m^2^), V is the DC voltage, and $\gamma_{AO}$ is the interfacial surface tension between the aqueous and oil phases (N/m). Figure 1c shows the off-state and Figure 1d shows the on-state in the pixel, where the oil moves into the corner of the pixel and the pixel substrate area becomes visible.

However, a colorful EWD presents many challenges, such as brightness, color gamut, and contrast ratio [11]. Most of the colorful EWDs, including one-layer [1,12] and three-layer architectures [2,13], still suffer from the common drawbacks of scalability and low reflection efficiency [11]. The main focus of one-layer colorful EWD studies is on dividing the area into individual RGBK (red, green, blue, and black) oil switches without a color filter, or using an RGB color filter with black oil. However, the resolution and oil dosing efficiency obtainable with individual RGBK oil switches approach is limited, since a full color pixel has four times the area of a monochromatic pixel, and individual RGBK color oils in one pixel can only be dosed by serial dosing techniques, such as ink-jet-printing (IJT). Furthermore, using an RGB color filter with black oil will reduce the brightness of the display due to light adsorption. For previous three-layer architectures, the complicated fabrication process is the drawback, since three separate substrates are assembled into one panel before sealing. 

In this paper, we present a full scalable fabrication and testing processes for multi-color segmented EWDs, with potentially unprecedented electro–optical performance. First, we briefly describe a three-layer architecture design of a colorful EWD. Next, a scalable fabrication process of EWDs, consisting of high-speed screen printing of fluoropolymer, slit-coating of photoresist, oil/water self-assembly, and subsequent integration of three independent layers into a complete colorful EWD by using accurate alignment and coupling equipment is investigated. Also, the electro-fluidic display inspector (EFDI) system for efficient and scalable testing processes is provided, the display parameters and defects are inspected just by plugging the monochromatic EWD into the test-jig for several seconds. Finally, the testing results of the colorful EWDs are presented using our proposed processes. The goal of this paper is to set up scalable and efficient fabrication and testing processes of colorful segmented EWDs, and thereby further expand the global research base supporting this new technology. The ease of a high-yield batch of colorful segmented EWDs is facilitated by utilizing the processes investigated herein.

## 2. Three-layer Architecture Design

**Figure 2:**
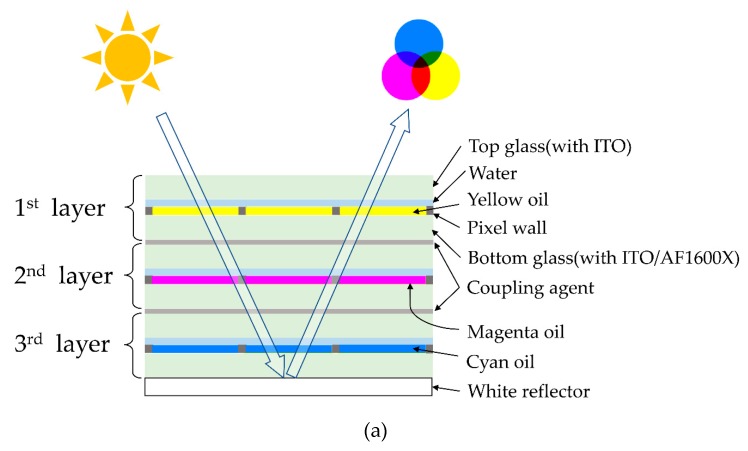

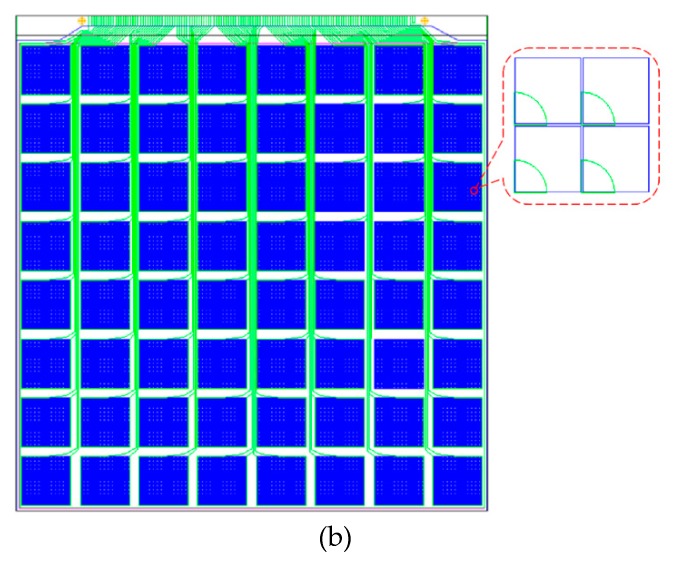
A three-layer colorful segmented electrowetting display design. (**a**) Side view of three-layer architecture pixels with the principle of subtractive color mixing in a reflective display. (**b**) Top view of the segmented electrowetting display arrays and details of pixel design, with the square pixel grid and notch-patterned indium tin oxide (ITO) electrode.

Figure 2a shows the design of the three-layer vertical stack architecture for a segmented colorful EWD. The color system for this reflective display is based on cyan, magenta, and yellow subtractive color mixing. The oil of each layer can be switched independently under the electric field between the top glass, with an indium tin oxide (ITO) electrode, and the bottom glass, with a patterned ITO electrode, to reflect different colors from the white reflector underneath the three layers. All three colorful layers are fabricated and tested by exactly the same processes, except for the color of the oil. Each layer consists of a bottom glass with a patterned ITO electrode, an amorphous fluoropolymer (e.g., Chemours AFX1600X, Wilmington, DE, USA) for the hydrophobic layer and dielectric [14,15,16], a negative photoresist grid for the pixel walls [17,18,19], the two fluids (polar water and nonpolar oil), and the top glass with the ITO common electrode. Alignment and coupling should be done by making use of accurate mounting equipment with a coupling agent to integrate the three layers together. The fabrication process to confine the oil film in each pixel has been described previously [20,21,22,23,24]. However, there are no full and scalable fabrication and testing processes for colorful EWDs, as detailed in the following sections. Figure 2b presents the top view of three-layer colorful segmented EWD design. The green lines are patterned ITO electrodes, and the square electrode design with a notch in the pixels are proven to be advantageous for oil switching [25]. The blue square grids are the negative photoresist pixel wall arrays. Detailed design parameters are listed in Table 1.

This architecture significantly increases the resolution of the display, because the size of the overall colorful pixel equals the subpixel of each layer in vertical direction. Moreover, if defects are found in any layer before assembly, only the corresponding layer needs to be replaced, instead of the whole assembly. Finally, there is less light loss and brilliant colors are achieved without a color filter.

## 3. Microfabrication

### 3.1. Materials

For materials, commercial indium tin oxide (ITO)-patterned glass (0.6 mm thick, 100 Ω/□ resistance) was used for the substrates (purchased from Leaguer Optronics Co., Ltd., Shenzhen, China). Amorphous fluoropolymer Teflon AF1600X were purchased from Chemours (Wilmington, DE, USA). The photoresist was a negative type modified epoxy, co-developed with a local material supplier. It can be developed in an aqueous solution (KOH) to facilitate industrialization. Colored oils were synthesized by our team, who provided cyan, magenta, and yellow colors. The flex-foil were designed in-house and manufactured by Xinteng Electronics Co., Ltd. (Shenzhen, China).

### 3.2. Fabrication Process

**Figure 3:**
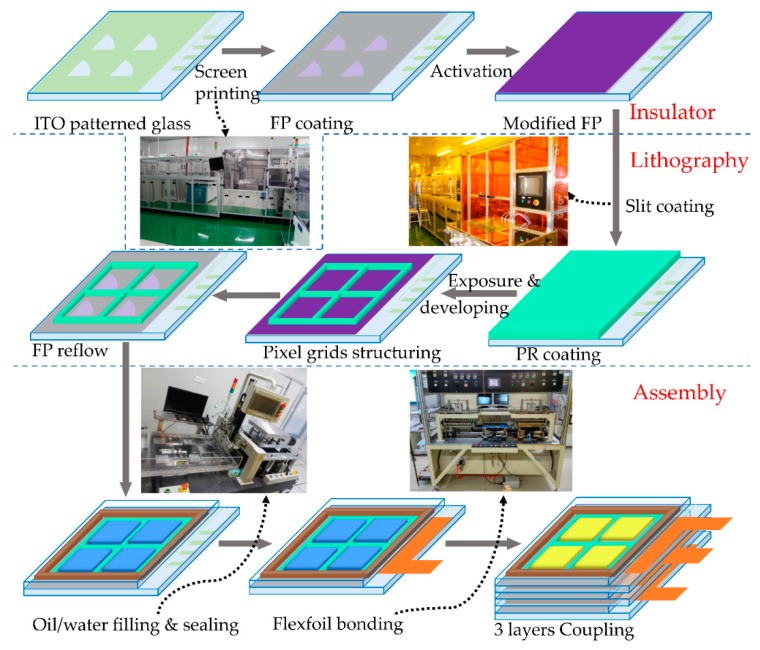
Schematic of scalable and efficient fabrication steps for a three-layer colorful segmented electrowetting display, including photos of some equipment.

The fabrication process of the colorful segmented EWD array is illustrated in Figure 3. We will describe herein the process and guidelines that provided robust, repeatable results using fabrication equipment available to most of those working in microfabrication or electronic devices. The G2.5 pilot manufacturing line designed by South China Normal University (SCNU) and Tsukishima Technology Maintenance Service Co., LTD (TTMS) (Chiba, Japan) was employed to achieve this scalable fabrication process. All the equipment of the G2.5 pilot was housed in a Class 10,000 environment, with the internal process areas maintained at Class 1000. The general steps required for the fabrication process can be divided into three main steps: insulator, lithography, and assembly.

The insulator step involved printing amorphous fluoropolymer (FP) for the insulator layer. Commercial ITO substrates were cleaned in an LCD cleaning line for G2.5 glass (400 mm × 500 mm). Amorphous fluoropolymer (FP) layers were coated using screen-print with a thickness of 600–1000 nm, followed by baking on hotplates at 100 °C to remove the bulk of the fluorocarbon solvent. Heating in an oven at a temperature of 185 °C for 30 min ensured that all solvent was removed. The cleaning line, screen-printer, hot plates, ovens, and developing line were supplied by Autech (Shenzhen, China). The hydrophobic fluoropolymer surface was then activated to be hydrophilic by reactive ion etching (RIE), using equipment supplied by the Institute of Microelectronics, Chinese Academy of Science (Beijing, China) to facilitate its subsequent coating by photoresist.

Next was the lithography step. Photoresist (PR) was coated onto the modified fluoropolymer surface via slit coating, using equipment supplied by TTMS. The thickness of the photoresist was uniform, in the range of 5 to 8 μm. A conventional lithography process was implemented to fabricate the photoresist layer into square pixel grids (200 μm × 200 μm). The exposure equipment (PA-4050-5K) in this step was supplied by Seiwa (Tokyo, Japan). Different patterns could be achieved by changing the mask design. After development of the PR, a thermal reflow process with a temperature of approximately 200 °C was used for 2 h to return the surface of the fluoropolymer back to its native hydrophobic state.

The final step of the fabrication process was assembly. Oil was filled by self-assembly technology, making use of its surface tension. The electrowetting substrate, as manufactured above, was slowly lowered through an oil film floating on water, using filling equipment developed in-house. Using a conventional screen printer, a UV-curable sealing agent was printed on the top ITO glass. This top-glass was then combined with the filled substrate glass and sealed using external pressure. 

Following this, ITO connection pads on the substrate were connected to a flexfoil (flexible printed circuit, FPC) by using a bonding machine (FPC-300, Shihao, Kaohsiung, Taiwan). After that, the three layers of monochromatic EWD panels (cyan, magenta, and yellow) were aligned and coupled together accurately, using a coupling agent and coupling equipment supplied by M-Triangel Technology Co., Ltd (Shenzhen, China).

## 4. Testing Process

**Figure 4:**
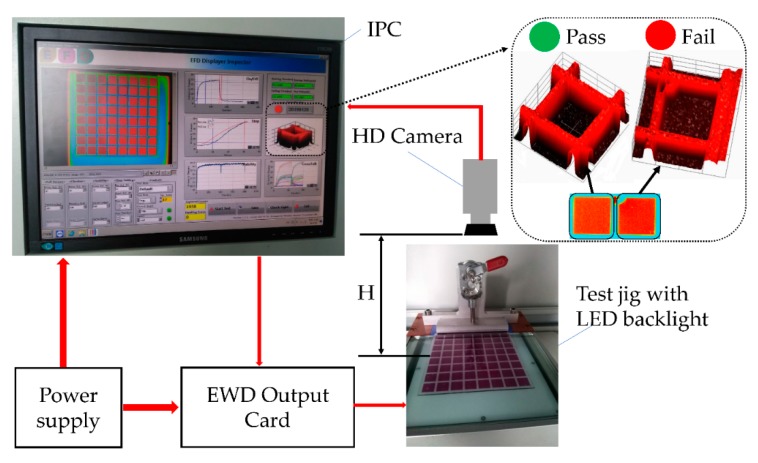
Schematic of the electro-fluid display inspector system for scalable testing process.

The testing process of monochromatic EWD screens should be implemented before the flexfoil bonding step of the fabrication process mentioned in the previous section, to ensure that no EWD screen with defects is put into the bonding process. Manual testing is time consuming, unstable, and difficult to operate—since many electro–optical parameters should be measured and defects should be found, it does not match scalable fabrication. However, the electro-fluidic display inspector (EFDI) system co-developed by South China Normal University (SCNU) and Fulitech Co., Ltd. (Shenzhen, China) in this paper was employed for automatic and industrial testing for large quantities of EWD panels. As shown in Figure 4, the power supply module provides AC 220 V to an industrial personal computer (IPC) and outputs DC +5 V to the EWD output card. The high-definition (HD) camera connected to IPC was fixed above the test jig with an LED backlight, which kept an appropriate height from the surface of the EWD panel to properly detect the light intensity of every EWD segment (from 1 to 64). A warning symbol was generated if visible defects were found by automatically processing 3D light intensity images of each segment (from 1 to 64). The dedicated test jig connecting the ITO pads on the EWD provided the voltage to switch each segment on and off and vary the light intensity of segments transported from the LED backlight. The IPC processed and analyzed the image captured by the HD camera. The EWD output card controlled by the IPC output signals and switched segments of the EWD panel to calculate the electro–optic response and detect defects in the EWD pixels. All the electro–optic response parameters (driving voltage, response time, and aperture ratio) and defects in the panel were obtained in 5 s by the software user interface in Figure 4, developed with LabVIEW (2017 SP1). The details in Figure 4 illustrate the comparison of the 3D light intensity images of pass (qualified) and fail (corner pixels missing) results in EFDI. Common defects and issues in the EWD panel found by the EFDI system included oil leakage, oil crossing the pixel wall, no closing, and un-filled pixels.

## 5. Results and Discussion

### 5.1. Fabrication Results

**Figure 5:**
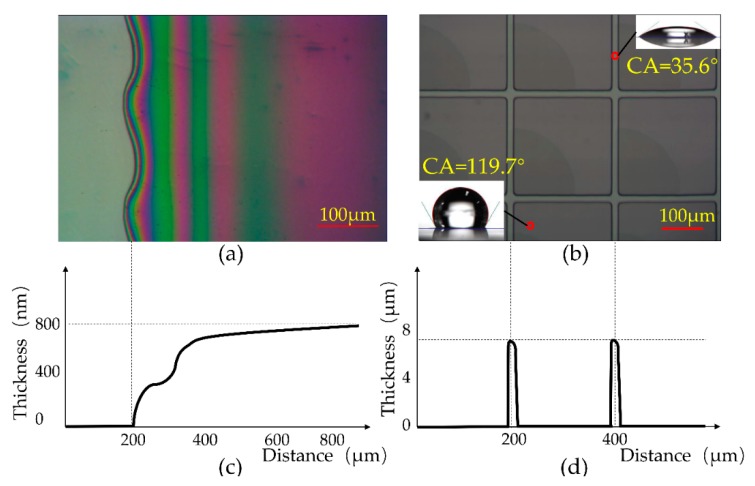
Fabrication results characterization after insulator and lithography steps. (**a**) Optical micrograph of the edge of the homogeneous amorphous fluoropolymer Teflon AF1600X after the insulator step. (**b**) A micrograph of the patterned photoresist pixel grids with a 119.7° water contact angle on the hydrophobic fluoropolymer and 35.6° water contact angle on the hydrophilic photoresist after the lithography step. (**c**) Surface morphology data corresponding to the edge of the fluoropolymer in (**a**), measured by the stylus profiler. (**d**) Surface morphology data corresponding to pixel grids in (**b**), measured by the stylus profiler.

**Figure 6:**
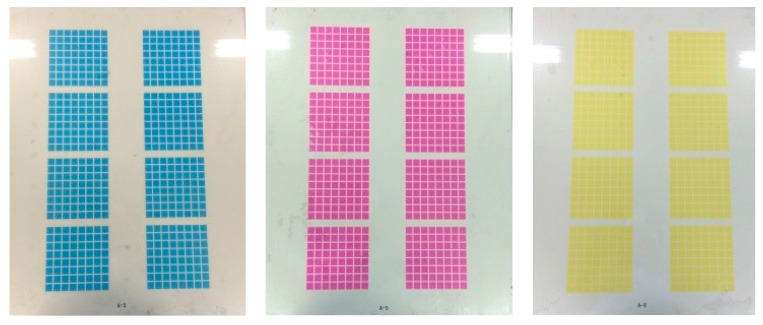
Photos of three color monochromatic segmented electrowetting display G2.5 size glass substrates after the oil/water filling and sealing of assembly step.

The electro–optical performance of the completed three-layer colorful EWD will be discussed in the next section. In this section, the fabrication results are briefly characterized, then several advantages of the scalable fabrication process described herein are reviewed and comparison with the conventional spin/dip coating. The fabricated substrate with patterned photoresist pixel grids after the lithography step is characterized according to Figure 5. The thickness of the films was measured by a stylus profiler (Dektak XT, BRUKER, Billerica, MA, USA). Figure 5a illustrates the optical microscope image of the FP, with a colored interference pattern due to variation in thickness at the edge of the FP. This area is used for measuring the thickness of the FP layer. Figure 5c shows the corresponding thickness of the FP, increasing from 0 to 800 nm, measured by the stylus profiler at the edge of the homogeneous FP film. Figure 5b shows the micrograph of the patterned photoresist pixel grids. The water contact angles of the surface in the pixel and the photoresist pixel grids are also illustrated. The water contact angle (CA) on the hydrophobic FP surface in the pixel exceeds 119.7°, while it was 35.6° on the hydrophilic photoresist pixel grid. Surface morphology data corresponding to pixel grids measured by the stylus profiler is shown in Figure 5d, we can observe a thickness of about 6.8 μm and 200 μm × 200 μm size pixel grids are obtained. All the results demonstrate both the FP layer and patterned PR layer were fabricated successfully by the combination of screen-printing and slit-coating processes. Figure 6 illustrates photos of three color (cyan, magenta, and yellow) monochromatic G2.5 size glass substrates (400 mm × 500 mm) after the oil/water filling and sealing process, which also demonstrates that we succeeded in implementing a scalable fabrication process of colorful EWDs with good efficiency and uniformity.

Compared with the normal fluoropolymer coating methods, such as dip or spin coating, screen printing is much faster. A 400 mm × 500 mm substrate can be coated with FP in 5 s, while there is a coating time of more than 65s for FP with spin coating. Besides the process efficiency, another advantage of screen printing is the high material utilization. Based on our experience, when spin coating a 6-inch square plate (150 × 150 mm) in our factory line with a thickness of approximately 800 nm FP, the material utilization efficiency will be 22%. In contrast, we have achieved a screen printing material utilization of FP of more than 80%, and this utilization can be expected to increase further depending on the number of consecutive coatings. A typical slit-coating PR time is 20 s per 500 mm plate. The average thickness deviation measured was 2%, measured in different areas of the PR, which shows good thickness uniformity of the photoresist layer, while there is a 65 s coating time and 5% thickness deviation with PR spin/dip coating per 50 mm plate. All parameters of screen-printing FP and slit coating PR in this paper compared with the conventional spin/dip coating are listed in detail in Table 2.

### 5.2. Electro–Optical Performance Testing Results

**Figure 7:**
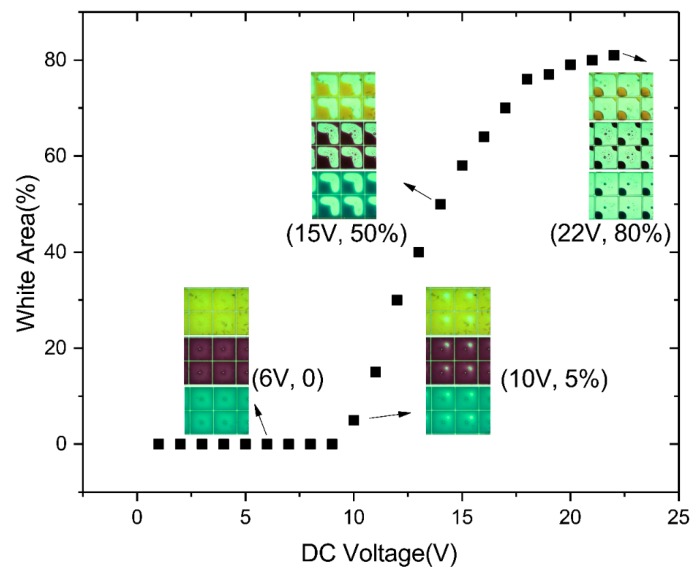
White area versus voltage curve for the fabricated three-layer colorful segmented electrowetting display, showing cyan, magenta, and yellow oil movement in pixels. Pixels are not activated from 0–9 V, threshold voltage for oil rupture is 10 V, ~50% aperture ratio is achieved at 15 V, and 80% aperture ratio at 22 V.

The electro–optical performance, including driving voltage, aperture ratio, switching speed, and color gamut is now briefly reviewed for a three-layer colorful segmented EWD, fabricated and tested using the scalable process described here. Electrowetting inherently provides an analog electro–optic response, which makes direct-drive segmented displays quite easy to implement by applying a DC voltage. The aperture ratio is used to measure the white area ratio (i.e., substrate percentage) in a pixel. It is defined as Formula (2):

(2) $$\mathrm{WA}\%=\left(1-\frac{A_{\mathrm{oil}}\left(V\right)}{A_{\mathrm{pix}}}\right)\times 100\%,$$

where $A_{\mathrm{oil}}$ and $A_{\mathrm{pix}}$ denote the area occupied by the oil and the overall area of the pixel, respectively, and *V* is the voltage applied to the device. As shown in Figure 7, white area variation was observed by microscope for a fabricated three-layer colorful electrowetting display under the application of a DC voltage from 0 V to 22 V. The design was of a notch area with no electrode occupying 20% of the pixel area, and all three layers of oil homogeneously covering the entire pixel when the driving voltage was below 9 V. Oil began to rupture when the driving voltage rose to 10 V, an approximately 50% aperture ratio was achieved at 15 V, and the oil was driven to the notch corner with 80% maximum aperture ratio when the voltage reached 22 V. 

**Figure 8:**
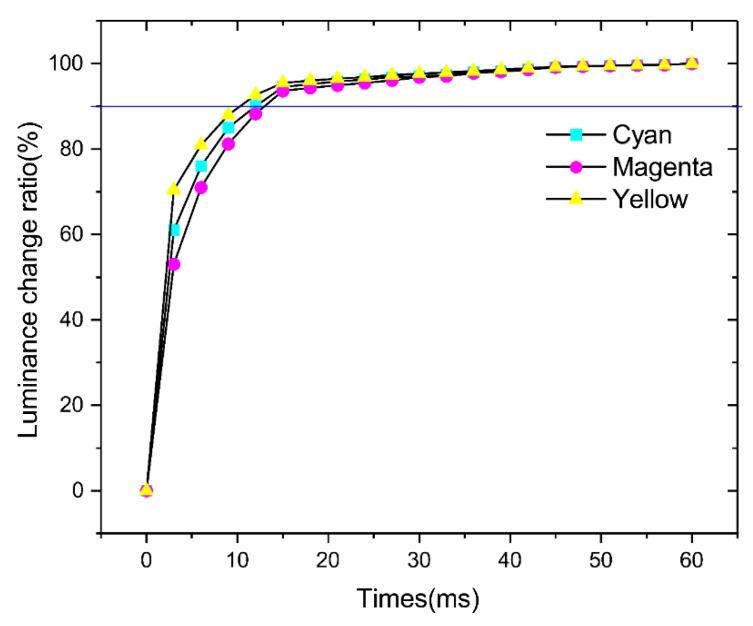
Pixel luminance change ratio due to oil motion as a function of time (switching speed) for each color layer (cyan, magenta, and yellow).

The switching speed of the three-layer colorful segmented EWD was measured using a 22 V driving voltage by using an optical colorimeter (Arges 45, Admesy, Ittervoort, The Netherlands). The switch-on time was obtained by plotting the luminance change ratio of the cyan, magenta, and yellow pixels with time, as shown in Figure 8. The luminance increased with time and reached saturation in 60 ms. The slight switching speed difference between the three colored oils was expected from their oil/water interfacial tension values. We defined 90% of the maximum luminance change as the effective display, and the average switching time of each layer was about 12 ms (for 90% of saturation level), significantly faster than EPD and in the video range.

**Figure 9:**
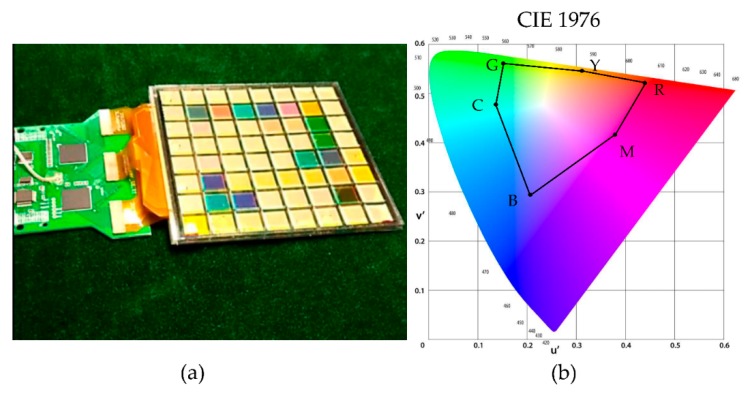
Electrowetting display color gamut demonstration, using scalable fabrication and testing processes. (**a**) A photo of a fabricated and tested three-layer segmented colorful electrowetting display. (**b**) Color gamut of the corresponding colorful segmented electrowetting display in (**a**), measured by an optical colorimeter (63% National Television Standards Committee (NTSC) in International Commission on Illumination (CIE) 1976).

Another important parameter for colorful display is color gamut. The colorful segmented EWD fabricated and tested by the described process has successfully shown a uniform paper like display, brilliant color (see Figure 9a), and 63% National Television Standards Committee (NTSC) color gamut in International Commission on Illumination (CIE) 1976 (see Figure 9b). We clarify that this color gamut is obtained by displaying a static image or a video with a frame switching time of more than 60 ms.

To improve the color gamut, the absorbance A of the oil has to be increased, as can be seen from the Beer–Lambert law:

(3) $$A=\log\left(\frac{1}{T}\right)=\varepsilon \cdot C\cdot t,$$

where T is transmittance, ε is the extinction coefficient (L/(mol·m)), C is the dye concentration (mol/L), and t is the oil layer thickness (m). From this equation, it is clear that either a higher dye concentration or a larger thickness increases A, but a thicker oil layer will require a larger driving voltage, therefore, the color gamut of our colorful EWD is focused on the balance between further dye concentration improvement and brightness.

## 6. Conclusions

This paper presented the scalable fabrication and testing processes for a three-layer colorful segmented EWD with a potentially unprecedented electro–optical performance. First, we briefly described a three-layer architecture design of a colorful EWD. Next, the scalable fabrication process of a three-layer colorful segmented EWD, consisting of a high-speed screen print fluoropolymer, slit-coating photoresist, self-assembly oil/water filling and closing, bonding, and coupling was introduced. Also, the electro-fluidic display inspector (EFDI) system for an efficient and scalable testing process was provided, the display parameters and defects were inspected just by successfully plugging the monochromatic EWD into the test-jig for 5 s. Finally, characterizing and listing advantages of the described fabrication process showed its uniformity, efficiency, and high quality. The results were then further described in terms of the electro–optic performance (DC 22 V driving voltage, 80% aperture ratio, 12 ms switching speed, and 63% NTSC color gamut were demonstrated). The main contribution of this work was to propose a full description of scalable processes from the substrate glass preparation of fabrication, to complete testing of a three-layer colorful segmented EWD by anyone skilled in microfabrication or displays, to quickly and successfully set up scalable fabrication and testing of colorful electrowetting displays, resulting in a high electro–optic performance. Future work may also pursue the use of similar processes for three-layer flexible colorful EWDs with thin film transistor (TFT) substrates and a high-yield batch of colorful EWDs with further electro–optic performance improvement. 

## Figures and Tables

**Table 1:** Design parameters of a colorful segmented electrowetting display (EWD).

Panel Size (mm)	Segments	Pixel Size (μm)	Photoresist Thickness (μm)	Pixel Wall Width (μm)	Colors	ITO (nm)	Fluoropolymer Layer (nm)
96 × 96	8 × 8	200 × 200	6.8	10	8	25	800

**Table 2:** Coating parameters of the fluoropolymer (FP) and photoresist (PR) in this paper versus spin/dip coating.

Parameters	Screen Printing FP/Slit Coating PR in This Paper	Spin/Dip Coating
FP coating time	5 s	65 s
Area of FP coating	400 mm × 500 mm	66 mm × 50 mm
Material utilization of FP	80%	22%
PR coating time	20 s	65 s
Deviation of PR thickness	2%	5%
